# Laparoscopic Repair of a Right Paraduodenal Hernia

**DOI:** 10.1055/s-0038-1667149

**Published:** 2018-07-20

**Authors:** John Mathew Manipadam, Lekha V., Vamsi Syamprasad, Ramesh H.

**Affiliations:** 1 Department of GI Surgery, VPS Lakeshore Hospital and Research Centre, Kochi, Kerala, India; 2 Department of GI Surgery and Liver Transplantation, VPS Lakeshore Hospital and Research Centre, Kochi, Kerala, India

**Keywords:** right paraduodenal hernia, laparoscopic repair

## Abstract

Laparoscopic repair of a right paraduodenal hernia has been described sparingly in literature. We present an account of how we laparoscopically repaired a right paraduodenal hernia along with a review of the current literature as regards the various techniques that have been attempted. With the patient in supine position, and with umbilical camera port and three 5 mm ports, we mobilized the cecum and ascending colon up to the third part of the duodenum, thereby widening the neck of the hernia sac in the Waldeyer fossa. This method is ideal for the less severe incomplete rotation presenting with right paraduodenal hernia where there are no Ladd's bands and there is no requirement for fetalization of the bowel.

Internal hernias constitute a miniscule of all the causes of intestinal obstruction varying from 0.2 to 5.8%. Paraduodenal hernias account for nearly half of all internal hernias, left being three times commoner than the right. 1 Laparoscopic repair of a right paraduodenal hernia has been described sparingly in literature. We present an account of how we laparoscopically repaired a right paraduodenal hernia along with a review of the current literature as regards the various techniques that have been attempted.

## Preoperative Preparation

**Fig. 1:**
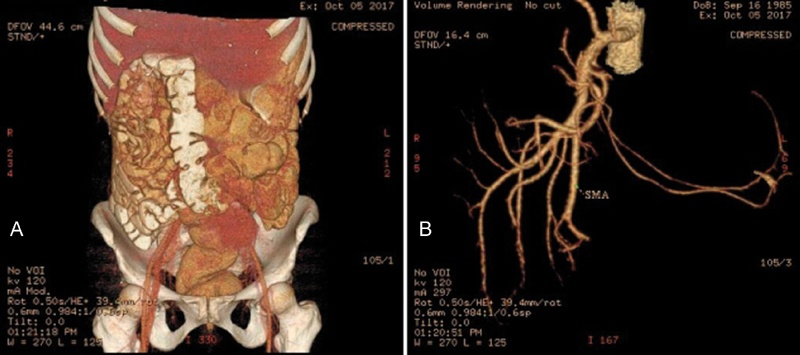
Computed tomography reconstruction images showing ( **A** ) clumping of small bowel loops in right subhepatic region ( **B** ) coursing toward right of corresponding superior mesenteric artery branches, suggestive of right paraduodenal hernia.

A 31-year-old lady presented to us with 2 months history of vague upper abdominal pain with bloating and occasional vomiting. She was evaluated initially with an upper gastrointestinal endoscopy which revealed only antral gastritis and was treated for the same with proton pump inhibitors. However, her symptoms persisted and she was evaluated with a computed tomography scan of the abdomen which revealed clumping of the proximal jejunal loops in the right subhepatic region with anterior displacement of the mesenteric vessels, with the majority of the proximal superior mesenteric artery branches coursing unusually to the right rather than left suggesting a probable right paraduodenal hernia ( Fig. 1 ). With this diagnosis in mind, she was posted for a diagnostic laparoscopy and proceed.

## Positioning of Patient and Ports

**Fig. 2:**
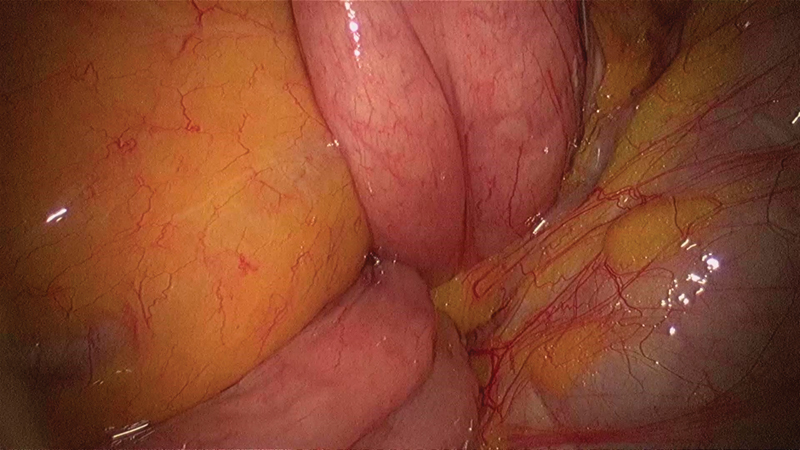
Small bowel loops trapped in the space behind the mesocolon of the ascending colon.

Patient was positioned supine with the operating and assistant surgeon on the left side of the patient with the monitor on the right. We used 10 mm umbilical camera port, one 5 mm working port in each iliac fossa for the right and left hand working ports and one 5mm port to retract the transverse mesocolon in the left hypochondrium. Intraoperatively, on performing a small bowel walk, we could see clumped small bowel loops trapped in the space below and behind the superior mesenteric vessels ( Figs. 2 3 4 ). We gently reduced the contents within this internal hernia by pulling out the entrapped small bowel. Once the entire bowel was pulled out, the defect underlying the mesentery could be seen clearly ( Fig. 5 ). The third part of the duodenum could be seen near the cephalad border of the defect and the superior mesenteric vessels above, similar to the description of the mesentericoparietal fossa of waldeyer 2 ( Fig. 6 ). To prevent further entrapment in this hernial defect, we widened the neck of this sac by mobilizing the cecum and ascending colon from lateral to medial reaching up to the third part of the duodenum ( Figs. 7 8 9 ). Thereby, the defect of the hernia became continuous with general peritoneal cavity excluding all possibilities of any future herniation. ( Fig. 10 )

**Fig. 3:**
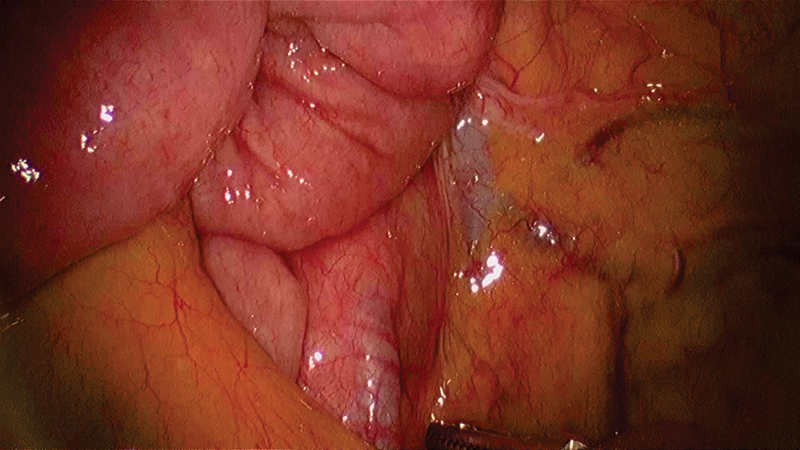
Small bowel loops entering the defect behind the mesocolon of the ascending colon in front of the central big vessels.

**Fig. 4:**
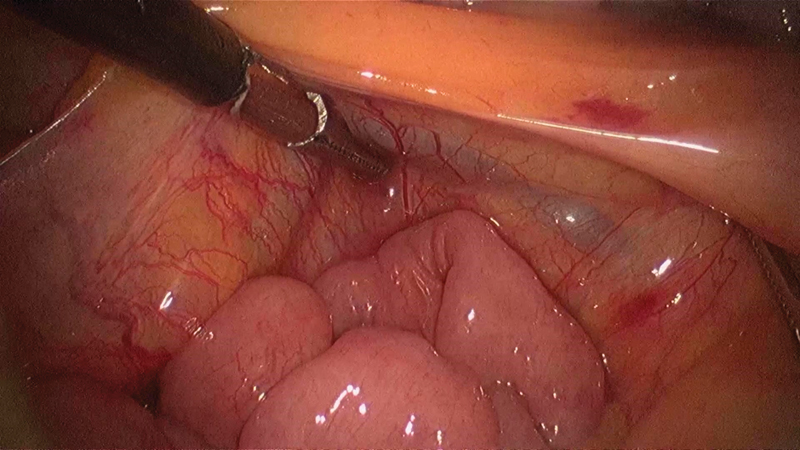
Small bowel loops entering the hernial sac behind the superior mesenteric vessels.

**Fig. 5:**
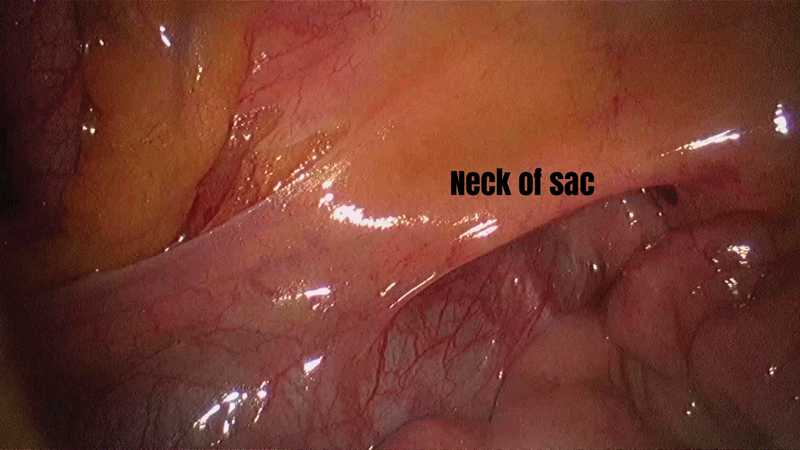
Neck of the hernial sac seen when small bowel loop contents have been reduced.

**Fig. 6:**
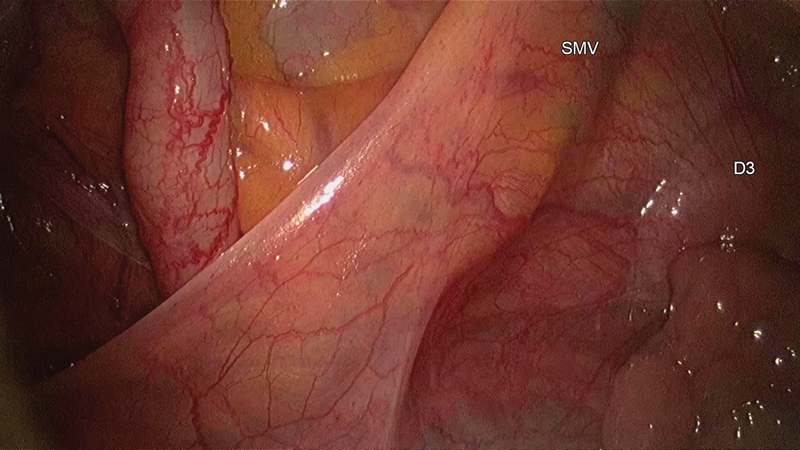
Third part of the duodenum (D3) and the superior mesenteric vessels bordering the defect seen on lifting the hernial sac with the working port instrument.

**Fig. 7:**
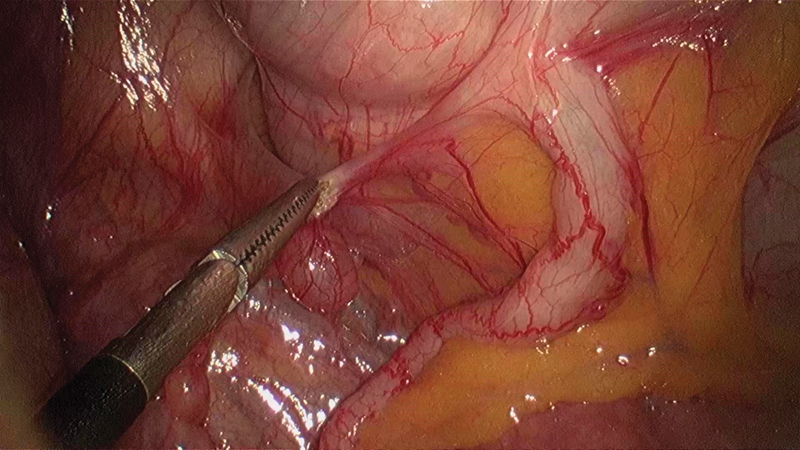
Mobilizing the cecum and ascending colon from right to left.

**Fig. 8:**
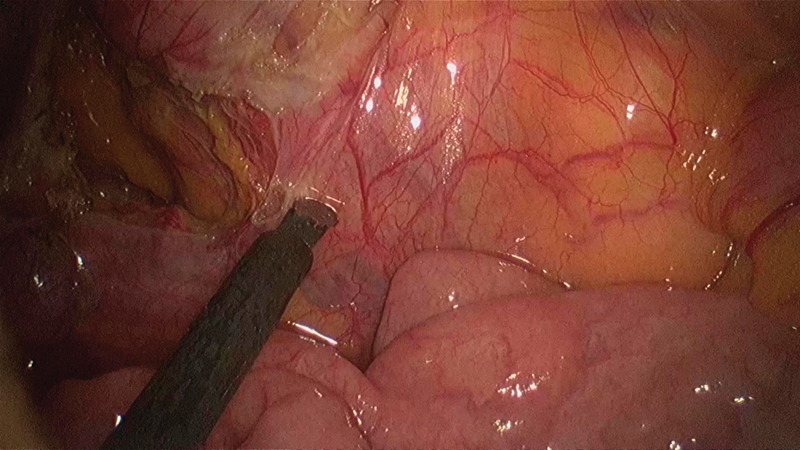
Mobilization of the ascending colon approaching medially.

**Fig. 9:**
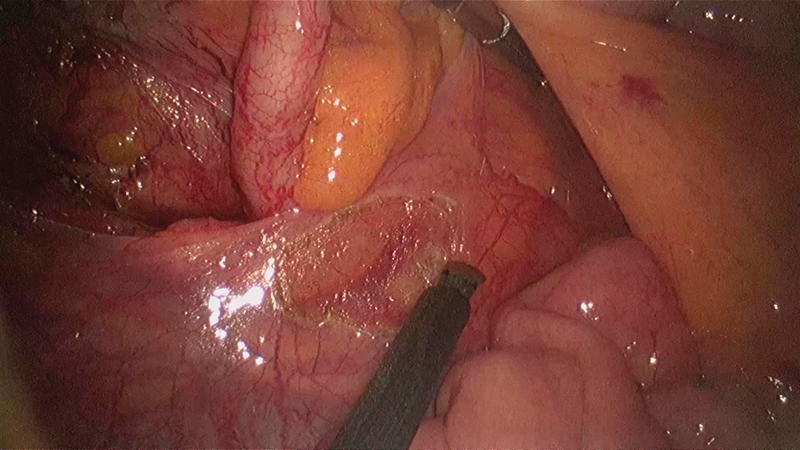
Incising the paraduodenal hernial sac wall during the final steps of the lateral to medial mobilization of the ascending colon.

**Fig. 10:**
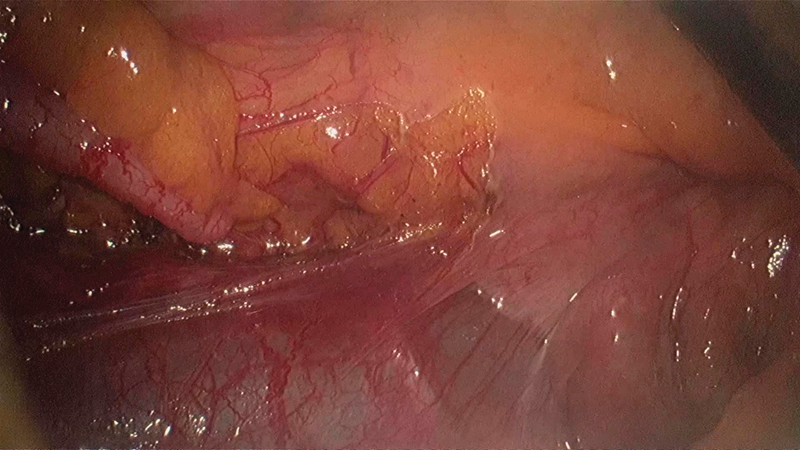
Hernial defect is continuous with the general peritoneal cavity on completion of the lateral to medial mobilization of the ascending colon up to the third part of the duodenum.

## Postoperative Care

Patient made an uneventful postoperative recovery. She was started on oral diet from postoperative day 1 and was discharged on normal diet the next day. At 3 months follow-up, she is doing well.

## Discussion

Right paraduodenal hernias develop when there is an abnormal incomplete rotation of the prearterial segment of the midgut. Instead of the usual 270 degrees anticlockwise rotation, the prearterial segment arrests at 9 O'clock position with 90 degree anticlockwise rotation alone. 3 The postarterial limb, however, continues in its normal rotation pattern and thus the cecum and ascending colon fuses to the parietes above the abnormal prearterial segment, thereby entrapping it in an internal hernia in the so-called fossa of Waldeyer.

There are eight reports of laparoscopic repair of right paraduodenal hernia in literature prior to ours.

Two were done in pediatric population that had severe incomplete rotation, which required a full fetalization of the bowel with division of Ladd's bands, straightening of the duodenum, and widening of cecocolic isthmus. 4 5 These patients presented with chronic abdominal pain and inability to eat well since childhood, suggesting an element of chronic partial intestinal obstruction. The symptoms thus correlated with the higher degree of incomplete rotation which was found intraoperatively. To achieve full fetalization of the bowel which is placing it in the position after the first stage of rotation, additional steps had to be performed that required considerable technical skill. These were hepatic flexure mobilization, detachment of omentum from transverse colon, division of Ladd's bands, mobilization of the duodenum, and so on.

In four of the other repairs, a simple release of the lateral attachments of cecum and ascending colon sufficed, thereby widening the hernia sac and releasing the small bowel entrapped in the sac. 3 6 7 8 These patients, similar to the one we reported, were adults who were asymptomatic in childhood and had symptoms of lesser duration compared with the pediatric group. This correlates with the intraoperative findings that there were no Ladd's bands or narrow jejunocolic isthmus along with the paraduodenal hernia. Thus, a simple release of the lateral attachments was adequate for postoperative recovery.

In two other laparoscopic repairs reported in literature, reduction in the contents of the sac was done, followed by closure of the defect with nonabsorbable suture. 1 9 There are two potential disadvantages of this technique. One is the close proximity of the superior mesenteric vessels during laparoscopic intracorporeal suturing, injury to which can have disastrous consequences. The other is the durability of the repair considering the fact that the tissues we approximate are flimsy. Therefore, we opted for the lateral to medial release, avoiding injury to the superior mesenteric vessels and chances of subsequent recurrence of herniation.

There are several factors that have influenced the outcome of this report as is obvious from the discussion. Age of the patient, duration of symptoms, severity of incomplete rotation, and technique of repair all have a bearing on the final outcome. In all these laparoscopic repairs, the duodenojejunal flexure location was found equally on either the right or the left side. This factor did not seem to significantly affect the outcome of the repair.

Our patient definitely belonged to the category of lesser degree of incomplete rotation and thereby benefitted from a simple release of the lateral attachments of the cecum and ascending colon with an umbilical 10 mm camera port and three other 5 mm ports. Our conclusion is that laparoscopic approach is definitely feasible in right paraduodenal hernia repair, but one must assess by preoperative imaging and intraoperative assessment the severity of the incomplete rotation to tailor the magnitude of the operation which can range from either a simple lateral mobilization of the ascending colon or closure of the hernia defect to a complete fetalization of the small and large bowel with division of the Ladd's bands. The new facts that emerge from this report are that right paraduodenal hernia presenting for the first time in adulthood with a short history may be associated with a lesser degree of incomplete rotation for which a simple lateral release of the attachments of the cecum and ascending colon would suffice. We would not recommend closure of the defect by intracorporeal suturing in laparoscopy due to the concerns earlier mentioned.

## References

[1] Tomino T Itoh S Yoshida D Right paraduodenal hernia successfully treated with laparoscopic surgery Asian J Endosc Surg 2015 8 01 87 90 25598063 10.1111/ases.12139

[2] Takeyama N Gokan T Ohgiya Y CT of internal hernias Radiographics 2005 25 04 997 1015 16009820 10.1148/rg.254045035

[3] Bittner J G IV Edwards M A Harrison S J Li K Karmin P N Mellinger J D Laparoscopic repair of a right paraduodenal hernia JSLS 2009 13 02 242 249 19660226 PMC3015939

[4] Dassinger M S Eubanks J W Laparoscopic repair of a right paraduodenal hernia in a child JSLS 2007 11 02 266 267 17761095 PMC3015731

[5] Gupta R K Kothari P Gupta A Laparoscopic management of right paraduodenal hernia along with the correction of malrotation in a pediatric patient: a case report Ann Pediatr Surg 2013 9 02 90 92

[6] Antedomenico E Singh N N Zagorski S M Dwyer K Chung M H Laparoscopic repair of a right paraduodenal hernia Surg Endosc 2004 18 01 165 166 10.1007/s00464-003-4516-2 14625766

[7] Theodorou A Lange J Saad S Case Report: Laparoscopic Treatment of a Right-Sided Paraduodenal Hernia [Internet] http:www.liebertpub.com/vor. 2013 [cited 2017 Dec 1]. Available from:http://online.liebertpub.com/doi/abs/10.1089/vor.2012.0098

[8] Palanivelu C Rangarajan M Jategaonkar P A Anand N V Senthilkumar K Laparoscopic management of paraduodenal hernias: mesh and mesh-less repairs. A report of four cases Hernia 2008 12 06 649 653 18465192 10.1007/s10029-008-0376-y

[9] Bhartia V Kumar A Khedkar I Savita K S Goel N Laparoscopic repair of a right para duodenal hernia J Minim Access Surg 2009 5 04 121 123 20407574 10.4103/0972-9941.59313 PMC2843129

